# Endocytic Motif on a Biotin-Tagged HIV-1 Env Modulates the Co-Transfer of Env and Gag during Cell-to-Cell Transmission

**DOI:** 10.3390/v13091729

**Published:** 2021-08-31

**Authors:** María Inés Barría, Raymond A. Alvarez, Kenneth Law, Deanna L. Wolfson, Thomas Huser, Benjamin K. Chen

**Affiliations:** 1 Facultad de Medicina y Ciencia, Universidad San Sebastián, Puerto Montt 5501842, Chile; maria.barriac@uss.cl; 2 Division of Infectious Diseases, Department of Medicine, Immunology Institute, Icahn School of Medicine at Mount Sinai, New York, NY 10029, USA; raymond.alvarez@mssm.edu (R.A.A.); kennethmlaw@gmail.com (K.L.); 3 Department of Physics and Technology, UiT The Arctic University of Norway, NO-9037 Tromsø, Norway; Deanna.Wolfson@uit.no; 4 Biomolecular Photonics, Department of Physics, Bielefeld University, 33615 Bielefeld, Germany; thomas.huser@physik.uni-bielefeld.de

**Keywords:** HIV-1, cell-to-cell transmission, virological synapses (VSs), HIV envelope, biotin acceptor peptide

## Abstract

During HIV-1 transmission through T cell virological synapses, the recruitment of the envelope (Env) glycoprotein to the site of cell–cell contact is important for adhesion and for packaging onto nascent virus particles which assemble at the site. Live imaging studies in CD4 T cells have captured the rapid recruitment of the viral structural protein Gag to VSs. We explored the role of endocytic trafficking of Env initiated by a membrane proximal tyrosine motif during HIV transfer into target cells and examined the factors that allow Gag and Env to be transferred together across the synapse. To facilitate tracking of Env in live cells, we adapted an Env tagging method and introduced a biotin acceptor peptide (BAP) into the V4 loop of Env gp120, enabling sensitive fluorescent tracking of V4-biotinylated Env. The BAP-tagged and biotinylated HIVs were replication-competent in cell-free and cell-to-cell infection assays. Live cell fluorescent imaging experiments showed rapid internalized cell surface Env on infected cells. Cell–cell transfer experiments conducted with the Env endocytosis mutant (Y712A) showed increased transfer of Env. Paradoxically, this increase in Env transfer was associated with significantly reduced Gag transfer into target cells, when compared to viral transfer associated with WT Env. This Y712A Env mutant also exhibited an altered Gag/biotin Env fluorescence ratio during transfer that correlated with decreased productive cell-to-cell infection. These results may suggest that the internalization of Env into recycling pools plays an important role in the coordinated transfer of Gag and Env across the VS, which optimizes productive infection in target cells.

## 1. Introduction

HIV-1 infection can spread from infected to uninfected T cells through adhesive contacts called virological synapses (VSs) [1]. This mode of HIV transmission is more efficient than cell-free virus infection [2,3] and is likely to influence viral spread and pathogenesis in vivo [4,5,6,7]. In this process, the cell surface envelope glycoprotein (Env) initiates cell–cell adhesion and leads to the recruitment of Gag and CD4 to the site of cell–cell contact in an actin-dependent manner [1,6]. Previous studies have followed the rapid movements of Gag to the VS after cell–cell adhesion using live microscopy [8,9]. Live imaging of the VS has revealed an ordered process whereby adhesion triggers the recruitment of Gag to the site of cell contact [8]. Gag recruitment to the synapse is rapid and occurs after Env-dependent cell–cell adhesion [3,8]. Additional cell adhesion proteins ICAM and ALCAM also facilitate adhesion and T cell polarization [10,11]. Recent studies indicated that Env appears to accumulate at low levels prior to recruitment of Gag [12]. The contribution of recycled vs. cell surface Env and the role of its path of recruitment to VS formation and infection are not well defined.

HIV-1 Env is a trimeric, type-I transmembrane protein that is synthesized through the endoplasmic reticulum (ER) and traffics through Golgi and secretory pathways prior to being packaged onto virus particles [13,14,15,16]. The gp160 precursor is cleaved to the surface gp120 and transmembrane gp41 subunits and traffics to the plasma membrane, where it is incorporated into new virus particles or rapidly recycled via endocytosis [17,18,19]. The rapid internalization of Env maintains low levels of Env on the cell surface, in order to avoid immune detection and evade infected cell clearance by antibody-mediated effector responses, such as antibody-dependent cellular cytotoxicity (ADCC) and antibody-dependent phagocytosis (ADP) [20,21]. The contribution of the newly trafficked vs. the recycled envelope in the formation of VSs is not known. The recycling of Env through endocytic recycling pathways through Rab11/Fip1c-dependent mechanisms is thought to be an important pathway that supports the specific incorporation of Env onto budding virus particles [19,22,23]. Additionally, the retromer complex, which is involved in retrograde endosome-to-Golgi transport, also has a role in Env incorporation [24]. A YXXL motif or dileucine motifs in the cytoplasmic tail of Env recruit the clathrin-dependent endocytosis machinery to initiate internalization of Env from the plasma membrane (PM) [17,25]. Mutation of YXXL diminishes the endocytosis of Env mediated by AP-2 and causes defects in cell-free virus infectivity [26], though it is not absolutely required for infection.

To better understand the role of HIV Env recruitment during VS formation, we adapted a previously described protein tagging method [27] to enable the tagging of HIV Env in order to visualize Env during VS formation and HIV-1 cell–cell transmission. The V4 loop of Env is flexible, accessible and sufficiently distant from functional domains, meaning that Env tolerates insertions without affecting the virus infectivity [28] or conformational dynamics of the glycoprotein [29]. Herein, we demonstrate that the insertion of a 15-amino acid biotin acceptor peptide tag (BAP) into V4 and biotinylation of this site do not reduce virus infectivity in cell-free and cell–cell infection assays. This Env-tagged virus was utilized in fluorescent imaging studies to monitor Env on the surface of infected cells, as well as Env within intracellular compartments.

Using the minimally perturbing BAP tag, we conducted studies to follow the fate of Env that is internalized in a YXXL-dependent manner and its impact on synapse formation, viral transfer and subsequent infection. Simultaneous tracking of WT Gag and Env during VS formation between CD4 T cells showed that Gag and Env co-localized at sites of cell–cell contact (VSs), but not within the cell at intracellular recycling pools containing Env. Live cell imaging studies with the WT virus indicated that Env traffics between intracellular compartments and the VS during cell–cell transmission. Studies with the Env endosomal recycling mutant (Y712A) virus showed the prominent accumulation of Env at the site of cell–cell contact, although with decreased Gag and increased Env translocation across VSs. The results may support a model for Env trafficking whereby Env internalization is coordinated with targeted trafficking and Env incorporation at the VS, which enhances HIV-1 infectivity during cell–cell transmission while maintaining a low surface density of Env.

## 2. Materials and Methods

### 2.1. Viral Constructs

All the plasmid constructs are based on the molecular clone pNL4-3 [30]. For the construction of HIV Env BAP-V4, pNL4-3 was modified by overlap extension PCR to insert the BAP motif GLNDIFEAQKIEWHE [22] after nucleotide 7441 in the V4 region of Env. This position is analogous to the HA11 tag from the study of Pantophlet et al. [31]. PCR fragments were introduced using NheI and BamHI into pNL4-3 and sequence verified. HIV Gag-iGFP, Gag-iCherry and Gag-iCerulean are infectious variants of pNL4-3 that have fluorescent proteins (GFP, mCherry and Cerulean, respectively) inserted between the matrix and capsid domains of Gag [3]. For the construction of V4 variants, HIV Gag-iGFP, Gag-iCherry and Gag-iCerulean were digested with NheI and BamHI to insert the Env carrying the BAP motif. The endocytic mutants BAP-V4-Y712A and BAP-V4-LL855A were generated by overlap extension PCR, and the fragment was inserted between NheI and BamHI and between BamHI and XhoI, respectively, and confirmed by sequencing. For the construction of Gag-iCherry BAP-V4/Y712A, the NheI/BamHI fragment containing the mutation was cloned. sshBirA and BAP control vectors [27] were a gift from Dr. Bakhos Tannous. Rab11-YFP construct was a gift from Dr. Walther Mothes [32].

### 2.2. Cells and Cell Culture

The human CD4 T cell line Jurkat (ATCC) was maintained in RPMI 1640, 10% fetal bovine serum (FBS), 100 units/mL penicillin and 100 μg/mL streptomycin. Human CD4 T cells were purified by negative selection using CD4 T cell isolation kit II (Miltenyi Biotec) from buffy coats obtained from blood donors from the New York Blood Center. CD4 T cells were activated with 5 μg/mL PHA-L and 50 IU/mL recombinant human interleukin-2 (IL-2) for 3–4 days. Jurkat T cells were nucleofected using program S-18 (Lonza). Jurkat and primary CD4 T cells were labeled at 37 °C for 5 min in 1 uM CellTrace Violet or DDAO Far Red (Invitrogen). J-BirA is a Jurkat cell line stably expressing the sshBirA enzyme by transduction with a pMSCV-BirA retrovirus. A clonal cell line, J-BirA clone 8, was selected for further experiments due to its higher capacity to biotinylate HIV Env BAP-V4. 293T cells (ATCC) were maintained in Dulbecco’s modification of Eagle’s medium supplemented with 10% Cosmic Calf serum (HyClone, Logan, UT, USA), 100 U/mL penicillin, 100 μg/mL streptomycin and 200 μM l-glutamine.

### 2.3. Preparation of Viral Particles and Western Blot Analysis

Viral particles were produced in 293T cells by standard calcium phosphate transfection [33]. After 16–18 h, post-transfection media were replaced with fresh media. The supernatant was harvested 2 days post-transfection, and the supernatant was centrifuged at 2000× *g* for 10 min at 4 °C to pellet cellular debris and syringe filtered through a 0.45 um pore filter. For Western blot analysis, the viruses were pelleted through a 1 mL 20% sucrose cushion by ultracentrifugation (Beckman Optima XL-100K Ultracentrifuge) using polyallomer tubes with an SW28 rotor (Beckman) at 28,000 rpm at 4 °C for 90 min. After pelleting, samples were lysed in RIPA buffer and denatured with 2× SDS loading buffer (Invitrogen). Samples were incubated at 90 °C for 10 min, and 10 μg of total protein determined by the Bradford method (or 2 μg p24 determined by ELISA) was run on 4–12% SDS NuPage (Invitrogen). Proteins were transferred to a PVDF membrane (Whatman) and immunoblotted using a 1/2000 dilution of anti-HIV AIDS patient serum (pooled neutralizing serum from 2 donors, AIDS Reagent Program, NIAID, NIH) and developed with goat anti-human IgG HRP (Jackson Immunoresearch) and chemiluminescence substrate (Pierce, Rockford, IL, USA). For the detection of the biotinylated envelope protein, anti-biotin HRP at 1/5000 dilution was used. Densitometry analysis was performed in image-J (version 1.42) using the gel analysis feature.

### 2.4. Cell-Free Infectivity Assay

The cell-free infectivity assay was performed as previously described [34]. Briefly, for infection studies, viral supernatants were quantified by p24 ELISA as previously described [3] and used to infect the target cells.

### 2.5. Cell-to-Cell Transfer Assay and VS-Mediated Infection Assay

The cell-to-cell transfer assay and VS-mediated infection assay were performed as previously described [34] with some modifications. For cell-to-cell transfer assays, target CD4 T cells were stained with violet cell proliferation dye (Invitrogen) and incubated with donor nucleofected (Amaxa Biosystems) J-BirA cells. Before co-culture, infected cells expressing Env-BAP were adjusted to 40–50% p24-positive cells by adding uninfected cells and stained with 20 ug/mL of either SA-Alexa Fluor 647, anti-biotin Alexa Fluor 488 or anti-biotin Alexa Fluor 647 at 37 °C for 1 h to label Env. Labeled cells were washed twice to remove any excess unbound Ab before incubation with target cells. For cell-to-cell transfer assays using HIV Env BAP-V4, Gag transfer was monitored by intracellular staining with anti-p24-PE or anti-p24-FITC (Beckman Coulter), or fluorescent protein was detected when using HIV Gag-iGFP, HIV Gag-iCherry or HIV Gag-iCerulean in the donor cells. Envelope transfer was monitored by anti-biotin Alexa Fluor 488 or anti-biotin Alexa Fluor 647 labeling after a 4 h co-culture.

For the VS-mediated infection, donor and target cells were co-cultured for 40 h and Gag expression was monitored by intracellular staining with anti-p24-PE or anti-p24-FITC (Beckman Coulter) in the case of HIV Env BAP-V4, or fluorescent protein expression was detected when using HIV Gag-iGFP, Gag-iCherry or HIV Gag-iCerulean. Env expression was determined by intracellular staining with anti-biotin Alexa Fluor 488 or anti-biotin Alexa Fluor 647.

### 2.6. Env Labeling

HIV Env BAP-V4, BAP-V4-fluorescent variants (Gag-iGFP, Gag-iCherry, Gag-iCerulean) or endocytic mutants (BAP-V4-Y712A and BAP-V4-LL855A) were nucleofected into J-BirA cells or co-nucleofected with BirA plasmid into Jurkat cells. Twenty hours after nucleofection, viable Jurkat or J-BirA cells were purified by centrifugation on a Ficoll-Hypaque density gradient. Forty-eight hours after nucleofection, cells were labeled at 4 °C or 37 °C for 1 h with 20 ug/mL of biotin labeling reagents (SA or anti-biotin antibodies with different fluorophores). Anti-envelope antibody b12 was used as a control Env stain detected with anti-human Alexa Fluor 647 antibody.

### 2.7. Fixed Cell Microscopy

To study HIV envelope co-localization with the BAP tag, J-BirA cells nucleofected with HIV Env BAP-V4 were stained with anti-envelope b12 monoclonal antibody (kind gift from Dennis Burton, Scripps Research Institute, La Jolla, CA, USA [35], and HIV Reagent Program, NIAID, NIH) followed by secondary anti-human Alexa Fluor 647 and anti-biotin Alexa Fluor 488.

For HIV Gag and Env visualization during cell–cell transfer, target CD4 T cells were labeled with violet proliferation stain (Invitrogen), and J-BirA cells were nucleofected with HIV Gag-iGFP, HIV Gag-iCherry or HIV Gag-iCerulean and incubated with SA-Alexa Fluor 647 or anti-biotin Alexa Fluor 488, respectively, at 37 °C for 1 h. To study Env and Rab11-YFP co-localization, J-BirA cells were nucleofected with HIV Gag-iCherry and Rab11-YFP, and Env was labeled with anti-biotin Alexa Fluor 647. The graph of the signal overlap and an ROI line to examine co-localization was created using ImageJ (http://rsb.info.nih.gov/ij, accessed on 24 September 2018). To study the VS transfer of the HIV Gag-iCherry BAP-V4/Y712A mutant, the wild type and tyrosine mutant were nucleofected into J-BirA cells, and the assay was performed as indicated above with some modifications. Target human CD4 T cells were stained with DDAO Far Red proliferation dye (Invitrogen), and donor cells were stained with violet proliferation dye (Invitrogen) followed by Env staining with anti-biotin Alexa Fluor 488. After co-culture, the cells Far Red+ Alexa Fluor 488+ were flow sorted and visualized by confocal microscopy to determine Gag and Env co-localization.

Fluorescence microscopy was conducted on a Leica SP5 DM microscope and DeltaVision Personal DV deconvolution microscope (Applied Precision). Deconvolution was performed using softWoRx 5.0.0. Images were acquired with an Olympus 60X/1.42 NA, with a 0.2 um z-step. Segmentation analysis on deconvolved image stacks was conducted using Imaris Bitplane. The number of puncta per cell was counted manually.

### 2.8. Monitoring Env Transfer by Live Cell Imaging

J-BirA cells were nucleofected with HIV Gag-iCerulean BAP-V4 and, after 48 h, labeled with anti-biotin Alexa Fluor 488 at 37 °C for 1 h, and after a washing step, cells were incubated with primary human CD4 T cells previously labeled with DDAO Far Red proliferation dye (Invitrogen). Live cell imaging was performed on a Zeiss Axio Observer Z1, Yokogawa spinning disk confocal microscope coupled with two Hamamatsu ImagEM EMCCD cameras to enable simultaneous imaging of Gag and Env fluorescent protein fusions. The specific acquisition settings were as follows: gain = 150, exp = 80 ms, 150 intervals every 4–5 s, 20 z-stacks, 0.75 um. Quicktime movies were generated from laser scanning confocal microscope file data using Metamorph software (Molecular Devices) and Imaris (bitplane) software.

### 2.9. Statistics

Statistical analysis of data was performed using Graph Pad PRISM software (GraphPad, San Diego, CA, USA). Significance between infected populations was calculated using a 2-tailed Mann–Whitney U test. Significance for multiple comparisons was performed using a one-way ANOVA corrected for multiple comparisons using Bonferroni’s correction. Biotin-Env/Gag ratio analysis was conducted with Imaris segmented images comparing relative fluorescence of biotin-Env-containing puncta. Values of p < 0.05 were considered significant. The mean ± SEM is shown in the graphs.

## 3. Results

### 3.1. Development of an Infectious Biotin Acceptor Peptide (BAP)-Tagged HIV Envelope Virus, HIV BAP-V4

**Figure 1:**
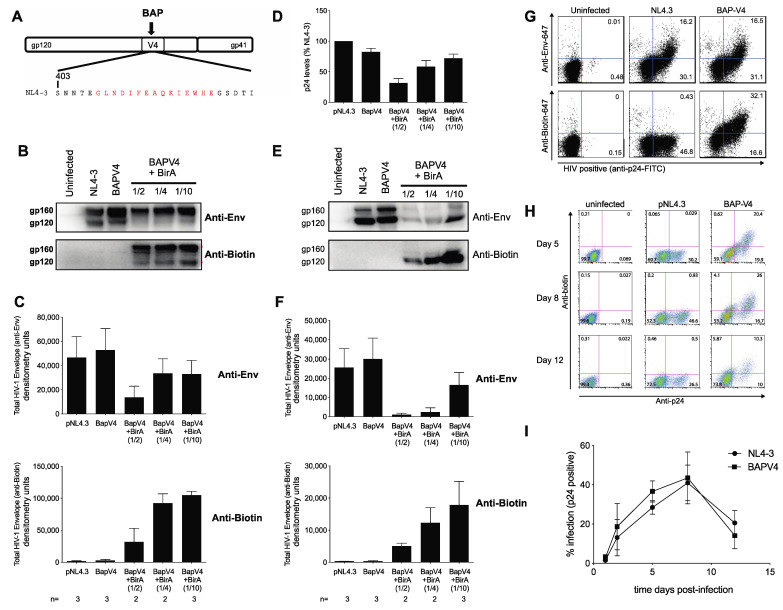
Development of HIV Env BAP-V4, a molecular HIV-1 clone expressing a biotinylated envelope protein. (**A**) Diagram of a biotin acceptor peptide (BAP) tag inserted into V4 region of NL4-3 Env glycoprotein. The amino acid sequence of the HIV NL4-3 gp120 Env V4 region indicating BAP insertion (red) and flanking HIV gp120 sequence (black). (**B**) Cell lysates from 293T cells transfected with HIV BAP-V4 were examined by Western blot with anti-Env (upper) and anti-biotin (bottom) antibodies. Cells were transfected with wild-type NL4-3 virus or HIV BAP-V4 and different ratios of Env/biotin protein ligase (BirA): 1:2, 1:4 and 1:10. (**C**) Densitometry of anti-Env (upper graph) and anti-biotin (lower graph) showing mean densitometry units from independent Western blots of transfected cell lysates. The number of repeat blots for each sample is shown (bottom). (**D**) Viral production from 293T cells transfected with wild-type virus or HIV BAP-V4 with and without different plasmid ratios of biotin protein ligase (+BirA 1:2, 1:4 and 1:10) was measured using p24 ELISA. The mean values of a triplicate replicate experiment are shown. Similar results were observed in three experiments conducted in triplicate. (**E**) Western blot of wild-type (pNL4-3) or HIV BAP-V4 viral particles purified through a 20% sucrose cushion. HIV BAP-V4 viral particles were produced in the presence of different plasmid ratios of biotin protein ligase (+BirA 1:2, 1:4 and 1:10). Western blots were probed with anti-biotin (upper panel) or anti-Env (bottom panel) antibodies. (**F**) Densitometry of anti-Env (upper graph) and anti-biotin (lower graph) showing mean densitometry units from repeat Western blots of virus particles. The number of repeat blots for each sample is shown (bottom). (**G**) Jurkat BirA (J-BirA) cells were spinoculated with 10 ng of cell-free WT HIV NL4-3 or HIV BAP-V4, and the levels of viral protein expression were assessed 48 h later. Flow cytometry plots show the levels of HIV Env and Gag p24 detected in uninfected (left panels), NL4-3- (middle panels) or BAP-V4 (right panels)-infected J-BirA cells. The levels of surface Env were detected using anti-Env b12 (top row) or anti-biotin antibody (bottom row), followed by intracellular p24 staining. (**H**) J-BirA cells were infected with 5 ng (p24 units) of cell-free HIV BAP-V4 virus by spinoculation, and the levels of productive infection were assessed by the level of intracellular Gag p24 accumulation at days 5, 8 and 12 post-infection. FACS plots show the levels of anti-p24 Gag staining and anti-biotin Env staining at days 5, 8 and 12 post-infection. (**I**) Graph shows the level of virus spread on days 2, 5, 8 and 12 post-infection. For all graphs, SEM of 3 experiments is shown.

To study Env trafficking patterns and the enrichment of the protein at cell–cell contact sites during VS formation, we adapted a protein tagging system that facilitates the fluorescent marking of HIV-1 Env with a small peptide insertion. To enable labeling of HIV-1 Env during HIV-1 infection, we inserted the 15-amino acid biotin acceptor peptide (BAP) into the V4 loop of gp120 within the context of a full-length infectious molecular clone of HIV-1 (HIV BAP-V4; Figure 1A). The BAP tag serves as a substrate for biotinylation through the E. coli biotin ligase BirA enzyme [27,36]. The BAP tag was inserted into the V4 loop of gp120, since this region has previously been shown to tolerate small insertions, without major impairment to virus entry [28].

First, we assessed the impact of the BAP-V4 tag on virus particle production and cell-free particle infectivity. 293T cells were co-transfected with the WT (pNL4-3) or the HIV BAP-V4 construct either without or with a BirA enzyme expression construct. A recombinant codon-optimized BirA enzyme with a signal sequence directing it to the secretory pathway (sshBirA) was used to direct an efficient biotinylation efficiency of the BAP tag on HIV Env [27]. HIV BAP-V4 virus production was comparable to that of WT HIV-1, as measured by p24 ELISA (Figure 1D). When HIV BAP-V4 was co-transfected with a 1:2, 1:4 or 1:10 ratio of the BAP plasmid to the HIV plasmid while maintaining the total DNA levels constant, the 1:10 ratio preserved the best HIV Env expression, while the lower HIV plasmid at the 1:2 or 1:4 ratio produced less virus (Figure 1B, top panels). In cell lysates of BAP-V4 Env, biotinylation was only observed in the presence of the BirA enzyme, while no biotinylation of WT Env was observed (Figure 1B,C, bottom panels). Biotinylation of BAP-V4 Env was observed at different ratios of the BAP-V4 and BirA plasmids, although a high ratio of the BirA plasmid to the HIV plasmid decreased Env expression (Figure 1B,C). A reduced signal for processed gp120 was observed in BirA co-transfected samples which may be indicative of inhibition of total Env, though gp120 was visible when a 1:10 ratio of BirA to BAP-V4 was employed (Figure 1B, Supplementary Figure S1). Virus particle production from the BAP-V4 construct was similar to WT HIV, though production was decreased when HIV BAP-V4 DNA was reduced relative to the BirA plasmid to express HIV that was biotinylated (Figure 1D). In transfected 293T cells Gag p55 and CA p24 expression of BAP-V4 construct was similar to WT when probed with polyclonal HIV patient IgG (Supplementary Figure S1). Examination of virus particles purified through a sucrose cushion showed that the biotinylated BAP-V4 Env was packaged onto virus particles and efficiently biotinylated at the 1:10 ratio of BirA to the HIV BAP-V4 plasmid in the transfection, although the co-transfected BirA plasmid appeared to limit the efficiency of Env packaging, especially at high ratios of BirA to the HIV plasmid (Figure 1E,F).

We tested the ability of the virus to infect a T cell line, Jurkat, that stably expresses a codon-optimized BirA enzyme, J-BirA cells. Newly infected cells could be measured by flow cytometry with both intracellular p24 staining or b12 monoclonal Ab anti-Env which gave rise to robust spinoculation of HIV BAP-V4 with a very similar fraction of infected cells relative to WT NL4-3 (Figure 1G). We speculate that the lack of an apparent infectivity defect or replication defect in the presence of BirA may be due to the lower levels of BirA expressed in the stable J-BirA cells. Anti-biotin antibody staining in these cells served as a very sensitive measure of cell surface Env with a higher fraction of infected cells staining with anti-biotin relative to the b12 monoclonal antibody staining (Figure 1G, bottom panel). Additionally, anti-biotin Abs bound to BAP-V4-infected cells and not WT HIV-1-infected cells, demonstrating that HIV-infected cells were biotinylated on the surface in a BAP-dependent manner (Figure 1G, lower panel).

J-BirA cells were infected via spinoculation with equivalent concentrations of WT HIV-1 or BAP-V4, normalized to the p24 content, and spreading infection was quantified by intracellular p24 staining at days 2, 5, 8 and 12 post-infection. BAP-V4 HIV-1 replicated with similar kinetics to WT HIV during this 12-day infection (Figure 1H,I). At peak replication (8 dpi), BAP-V4 yielded 47.3 ± 4.98% vs. 40.3 ± 4.22% for WT NL4-3, as measured by intracellular p24 staining (Figure 1I). The BAP-mediated biotinylation of HIV-1 Env in V4 does not impair the spread of HIV BAP-V4 relative to the wild-type virus. Any observed changes in processing efficiency observed in 293T co-transfection did not correlate with a negative impact on the Env function when infections were performed in cells stably expressing the BirA enzyme.

### 3.2. Live Cell Labeling of Surface Biotin in HIV BAP-V4-Infected Cells Enables the Measurement of Env Transfer during Cell–Cell Infection

**Figure 2:**
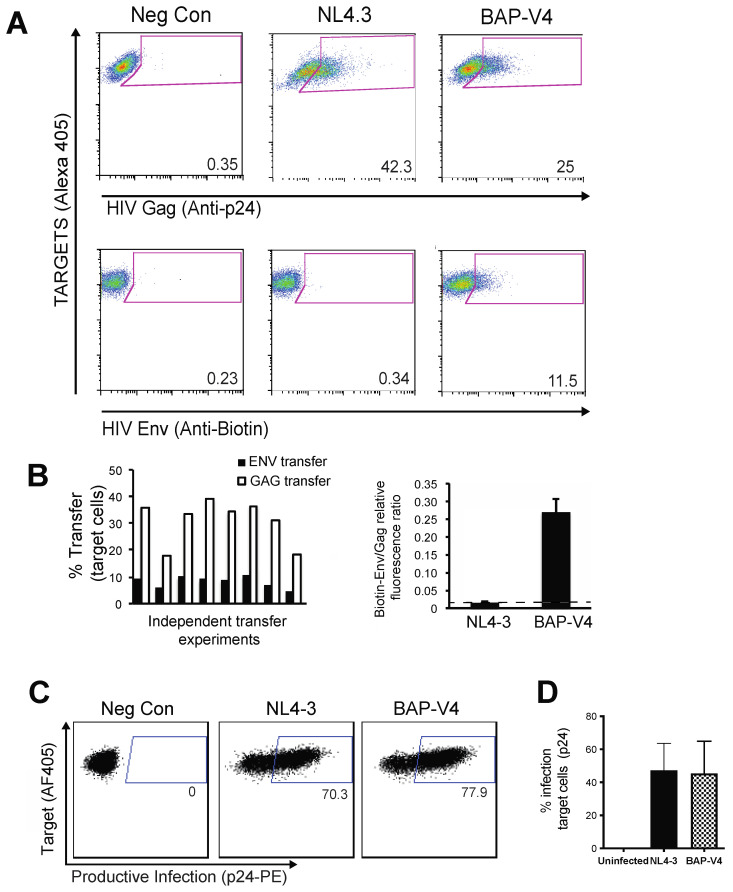
HIV Env BAP-V4 with a biotinylated envelope mediates infectious cell–cell transfer and transmission. Jurkat cells were nucleofected with WT, or BAP-V4 with BirA. All populations were then incubated at 37 °C with anti-biotin-647, washed and used as infected donor cells in 4 h cell–cell transfer assays with uninfected primary CD4 T cells. The levels of HIV transfer were measured by intracellular p24 staining. (**A**) Representative FACS plots show the level of HIV Gag (anti-p24) (top row) and Env (anti-biotin) (bottom row) transfer into primary CD4+ cells. (**B**) Graph on the left represents eight independent transfer experiments showing the Env and Gag transfer into primary CD4 T cells after cell–cell transfer assays as measured by anti-biotin and p24 intracellular staining. Graph on right is the cumulative mean ratio of biotinylated Env/Gag transferred into target cells using WT NL4-3 or BAP-V4 across all eight experiments. NL4-3 Env transfer level (which is not biotin labeled) represents background fluorescence measured in this assay. (**C**) Flow cytometry plots showing the level of virus infection measured by intracellular p24 staining (productive infection) in primary activated CD4 T cells 48 h after co-culture with uninfected (left panel), NL4-3 (middle panel)- or BAP-V4 (right panel)-infected cells. AZT was added 6 h post-co-culture in cell–cell transfer assay to limit the infection to a single round. (**D**) Graph shows the SEM levels of productive infection at 48 h after cell-to-cell infection, from *n* = 3 experiments.

To study the ability of HIV BAP-V4 to engage in cell-to-cell transfer through the VS, J-BirA cells were nucleofected with WT or BAP-V4 HIV-1 expression vectors and used as infected donor cells in cell–cell transfer assays [37]. To track biotinylated Env in cell–cell infection assays, HIV BAP-V4- and HIV WT-infected J-BirA cells were cultured in the presence of fluorescently conjugated anti-biotin Abs for 1 h at 37 °C. Cells were then washed and co-cultured with CellTrace Violet-labeled primary activated CD4 T cells for 4 h (Figure 2A). We observed that the level of Gag p24 transfer using HIV BAP-V4-infected cells was similar to WT NL4-3, as determined by p24 intracellular staining (Figure 2A, upper row). Biotinylated Env transfer into CD4 T cells was detected in co-cultures when HIV BAP-V4-infected cells were used as donors, but not with NL4-3-infected cells, which are not detected by the biotin antibody (Figure 2A, lower row). We observed a consistent percentage of cells that became Env- and Gag-positive after co-culture with infected cells indicative of transfer of both Gag and Env into the target cells (Figure 2B). As the HIV BAPV4 clone maintained its infectivity, we could also measure the level of productive infection after cell–cell transfer by staining for intracellular p24 of target primary CD4 T cells at 48 h post-co-culture. Staining of intracellular p24 at 48 h in infected cells was much more intense than the signal obtained following viral transfer (Figure 2C). The efficiency of transmission for WT NL4.3 HIV was similar to the percentage of productively infected cells produced by HIV BAP-V4 across multiple experiments (Figure 2D). This demonstrates that biotinylation of Env did not interfere with VS formation and that BAP tagging enabled the simultaneous quantification of Env and Gag across infectious VSs.

### 3.3. Visualization of HIV Gag and Env during VS Formation Using a Dual Fluorescent HIV-1 Gag-BAP-V4 Env

**Figure 3:**
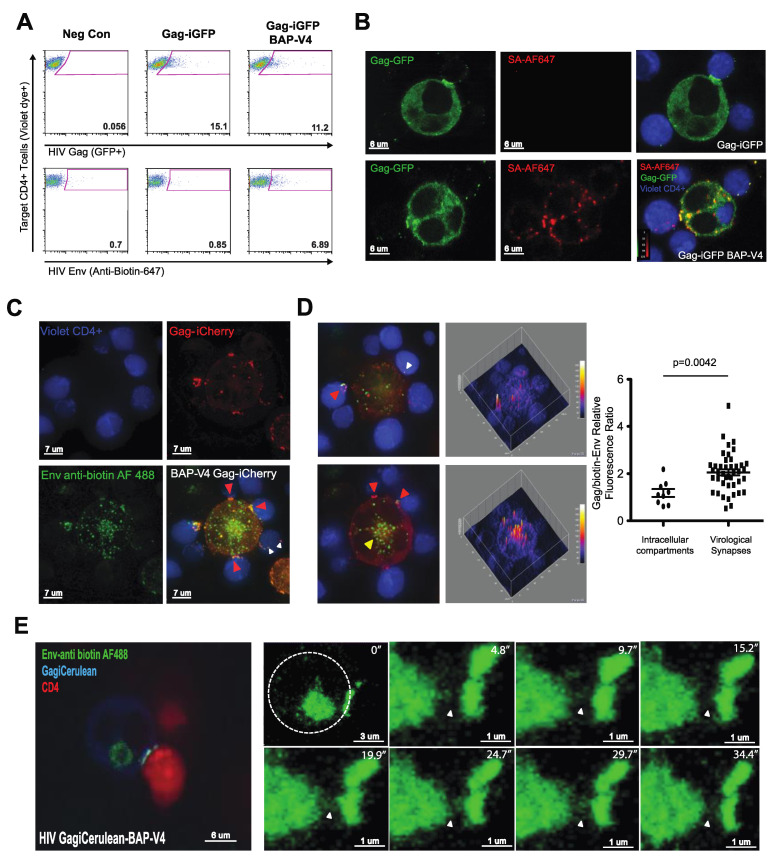
Simultaneous visualization of HIV Gag and BAP-V4 Env during virological synapse formation and cell–cell transfer. J-BirA cells infected with HIV Gag-iGFP or Gag-iGFP-BAP-V4 were used as donor cells in 4 h cell–cell transfer assays with primary CD4 T cells (Violet dye+; Alexa 405). (**A**) Flow cytometry dot plots show the levels of HIV Gag (GFP+; top row) and biotinylated Env (anti-Biotin-647; bottom row) transfer into target cells after 4 h transfer assays. (**B**) Laser scanning confocal image analysis of HIV-1 cell–cell transfer between Jurkat + BirA cells infected with Gag-iGFP (top row) or Gag-iGFP-BAP-V4 (bottom row) and uninfected primary CD4+ T cells (Violet+), with streptavidin Alexa Fluor 647 used to detect biotin. Left column shows localization of Gag-GFP. Middle column shows localization of Env (anti-Biotin-647). Far righthand column shows merged co-localization of Gag and Env at VSs. (**C**) Higher-resolution maximum-intensity projection images of Gag and Env co-localization in infected donor–target conjugates were generated using deconvolution microscopy performed on 20 medial z-stacks. Red arrowheads indicate the co-localization of Gag (mCherry) and Env (Alexa Fluor 488) at VSs. White arrowheads represent the transfer of Gag and Env into the target CD4 T cells. Ratio of Gag/Env fluorescence intensity (not reflective of molar ratio) was measured in intracellular compartments (*n* = 9) as compared to VSs (*n* = 43). (**D**) Representative image analysis of deconvolved images (left column) for quantifying the 3D intensity profiles of Gag and Env across z-stacks (right column), in order to compare the biotin-Env/Gag relative fluorescence ratio within intracellular compartments vs. at the VS. The yellow arrowhead represents intracellular compartments containing Env. Red arrowheads indicate the co-localization of Gag and Env at the VS. Graph displaying the Gag-to-Env relative fluorescence ratio within intracellular compartments as compared to the VS. (**E**) Spinning disk confocal image of Jurkat + BirA cells infected with Gag-iCerulean-BAP-V4 co-culture with Far Red dye-labeled primary CD4+ T cells. The image shows an infected cell engaged in virological synapse with a primary CD4 T cell. Image series depicts the 3D acquisition of Z-projection images showing the movement of HIV Env moving back and forth from the VS; numbers represent the time in seconds. White arrow in each frame points to the area where fluctuations in the biotin-Env signal are seen over time, more so than at other sites in the cell. See accompanying Supplementary Movie S1. Imaging settings for movie: gain = 150, Exp = 80 ms, 3D images were acquired every 4–5 s, with 150 total timepoints acquired over approximately 10 min. The medial 20 z planes were used for each 3D image, with 0.75 um z-increment between planes.

To study the relationship between Env and Gag recruitment at the VS, we constructed several viruses that contained the BAP-V4 Env tag expressed in cis with a fluorescently tagged Gag, which were termed HIV Gag-iCerulean-BAP-V4, Gag-iGFP-BAP-V4 or Gag-iCherry-BAP-V4. We first tested whether these dual Env and Gag-labeled viruses mediated HIV-1 transfer in 4 h cell-to-cell viral transfer assays with activated primary CD4+ T cells. We observed efficient Gag transfer with both Gag-iGFP WT and Gag-iGFP-BAP-V4 viruses as measured by flow cytometry (Figure 3A). Env transfer into target cells was detected by anti-biotin labeling of Gag-iGFP-BAP-V4-infected co-cultures. The specificity of biotinylated Env labeling was further verified by static confocal microscopy using anti-biotin Alexa Fluor 647 (data not shown) or streptavidin (SA) 647 in Gag-iGFP- vs. Gag-iGFP-BAP-V4-infected J-BirA cells (Figure 3B). The imaging study showed that anti-biotin and streptavidin labeling was only detected in Gag-iGFP-BAP-V4- but not Gag-iGFP-infected cells, which do not express the BAP-tagged Env. These experiments demonstrate that biotinylated Env can be simultaneously tracked with fluorescently tagged Gag during cell-to-cell transfer across VSs.

We next examined the localization of Env and Gag within infected cells during VS formation. J-BirA cells were infected with HIV Gag-iCherry-BAP-V4 and pre-labeled with anti-biotin Alexa Fluor 488 Ab at 37 °C for 1 h. Cells were then washed and co-cultured with primary violet-labeled CD4 T cells for 4 h (Figure 3C). The 3D intensity profile analysis of deconvolution microscopy images of HIV Gag-iCherry-BAP-V4-infected cells revealed that Env localized within intracellular pools and at the plasma membrane of infected cells. At the PM, Env could be observed to localize with Gag at VSs, while Gag did not co-localize as strongly at intracellular pools containing Env (Figure 3D). A relative fluorescence ratio of Gag/biotin Env at VS or at non-VS internal compartments was obtained by segmentation of Env at VS at these sites, and the relative fluorescence values were calculated at each site. We note that these values do not represent a molar ratio but can indicate potential spatial differences in the ratio of the two proteins.

To monitor Env trafficking during VS formation and virus transfer, we conducted live cell fluorescent imaging using dual-camera spinning disk confocal microscopy (Supplemental Movie S1). J-BirA cells were infected with HIV Gag-iCerulean-BAP-V4 and Env labeled with anti-biotin Ab (488) prior to co-culture with primary CD4+ T cells. During live imaging of co-cultures, we detected several VSs where Gag and Env polarized toward the interfaces of infected and uninfected cells. In these VS examples, we observed that intracellular pools of Env were located adjacent to VSs (Figure 3E). To study the role of these intracellular Env pools, we examined the movement of Env in reference to these intracellular pools during VS engagement. A time series of selected 3D reconstructions/time-lapse images from spinning disk images shows activity between intracellular pools of Env between an intracellular compartment and a VS, consistent with the exchange in Env from the intracellular pools to the VS and back (Supplemental Movie S1 and Figure 3E). Although the spatial and temporal resolution of the imaging did not permit tracking individual Env-containing puncta, these images provide some qualitative measure that surface-labeled and internalized Envs traffic near the site of VS formation.

### 3.4. Mutation of a Membrane Proximal Endocytosis Motif Decreases Productive HIV-1 Infection through Cell–Cell Transmission

To examine the role of endocytosis of Env on HIV cell–cell transfer and VS formation, we utilized two HIV-1 mutants carrying point mutations in the cytoplasmic tail of HIV Env gp41. These mutants have been shown to inhibit the endocytic recycling of Env from the surface of infected cells [26]. A tyrosine-to-alanine mutation within the membrane proximal YXXL motif at amino acid position 712 (pNL4-3) was shown to disrupt the interaction of Env with the μ subunit of AP-2, preventing efficient Env recycling from the surface of infected cells. In a similar manner, the C-terminal dileucine motif at amino acid position 855 (pNL4-3) was previously reported to inhibit Env endocytosis from the surface of infected cells when mutated to di-alanine, through inhibiting its interaction with AP-1 [25]. The two endocytosis mutants were cloned into a Gag-iGFP-BAP-V4 background to generate two endocytosis-mutant Env viruses.

**Figure 4:**
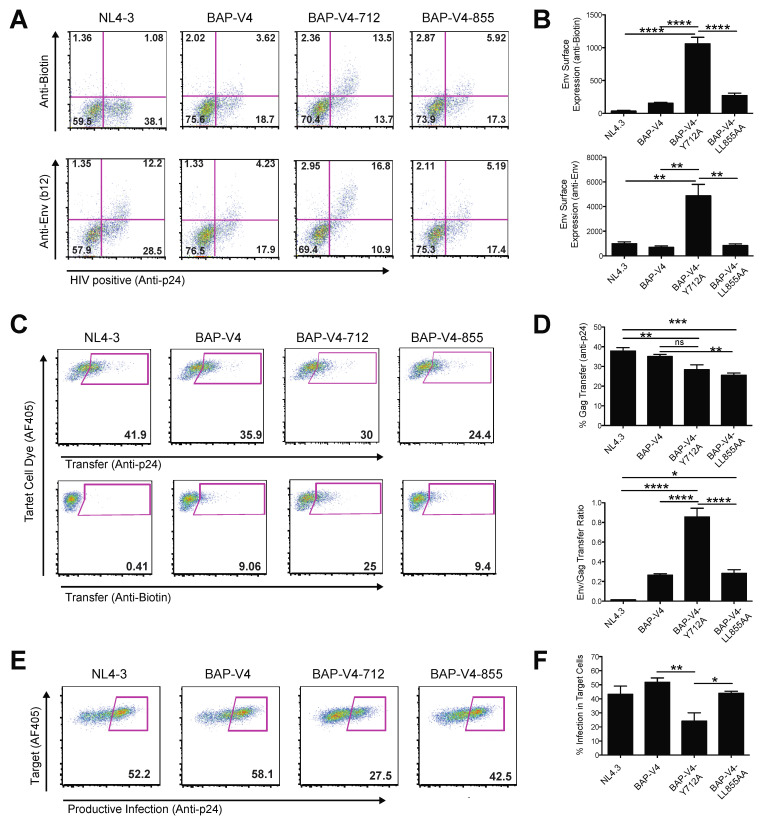
Endosomal recycling pools of Env support the coordinated cell–cell transfer of Gag and Env to promote productive infection across VS. J-BirA cells were infected with a series of HIV point mutant viruses (Y712A; LL855AA) that inhibit Env endocytosis/recycling from the surface of infected cells and which expressed the Bap tag in the V4 loop. (**A**) HIV-infected cells were labeled with anti-biotin Ab at 37 °C followed by intracellular Gag quantification using an anti-p24 Ab. FACS plots show the level of Env surface expression on the surface of pNL4-3-, BAP-V4-, BAP-V4-Y712A- and BAP-V4-LL855AA-infected J-BirA cells as quantified by anti-biotin antibody (top row) or the b12 anti-Env antibody (bottom row) and intracellular staining with a p24 anti-Gag Ab. (**B**) Graphs depict the MFI index (percentage of double positive cells multiplied by APC MFI) of the surface expression using anti-biotin (upper) or b12 antibody (bottom). The SEM of *n* = 3 experiments is shown. (**C**) HIV-infected and anti-biotin-labeled cells were used in 4 h cell–cell transfer experiments with violet dye-labeled primary CD4 T cells. The upper row of FACS plots depicts the level of Gag transfer (anti-p24, upper row) and Env transfer (anti-biotin-647, bottom row). (**D**) Upper graph represents the percentage of Gag p24 transferred into target CD4+ cells. Lower graph is the ratio between the Env transferred in the target cells and p24 in the donor cells. Graphs represent the SEM of *n* = 5 experiments. (**E**) Flow plots depict the level of p24 intracellular staining in primary target CD4 T cells 48 h after co-culture with HIV-infected donor cells. AZT was added after 6 h of cell–cell contact to limit the infection to a single round. (**F**) Graph shows the average level of productive infection in co-cultured target cells, as assessed by intracellular p24 staining. All donor cells were normalized to 40–50% p24-positive for cell–cell experiments. The SEM of *n* = 3 experiments is shown. Significance was assessed using a one-way ANOVA corrected for multiple comparisons using Bonferroni’s comparison test. * *p* ≤ 0.05, ** *p* ≤ 0.01, *** *p* ≤ 0.001, **** *p* ≤ 0.0001.

We examined if the Env Y712A and Env LL855AA mutations affected the accumulation of Env on the surface of infected cells. J-BirA cells were infected with BAP-V4-WT, Y712A or LL855AA, and the levels of surface Env expression were quantified using the anti-Env Abs b12 or anti-biotin Abs and analyzed via flow cytometry (Figure 4A). Env expression was normalized to intracellular Gag expression quantified by anti-p24 Ab staining. Analysis of surface Env expression showed an Env (b12)/p24 ratio of 0.2, 0.6 and 0.25 for BAP-V4 WT, Y712A and LL855AA, respectively (Figure 4A). In comparing the anti-biotin vs. p24 staining fluorescence intensities, we observed a similar phenotype for BAP-V4, Y712A and LL855AA with 0.18, 0.5 and 0.26 Env (anti-biotin)/p24 ratios, respectively (Figure 4A). Since the level of Env expression in a population of cells is measured not only by the proportion of cells expressing Env but also by the density of Env expressed on infected cells, we calculated an Env expression index that accounted for the frequency and MFI of Env surface expression. In doing so, we observed a significant 7-fold increase in Env surface expression in Y712A-infected cells vs. the WT virus, as detected by both anti-Env (b12) and anti-biotin antibodies (Figure 4B). Additionally, BAP Y712A-infected cells expressed 6-fold higher surface Env levels, as compared to BAP LL855AA-infected cells (Figure 4B). This indicates that BAP Y712A yielded the highest levels of surface Env accumulation as compared Env WT or Env LL855AA.

**Figure 5:**
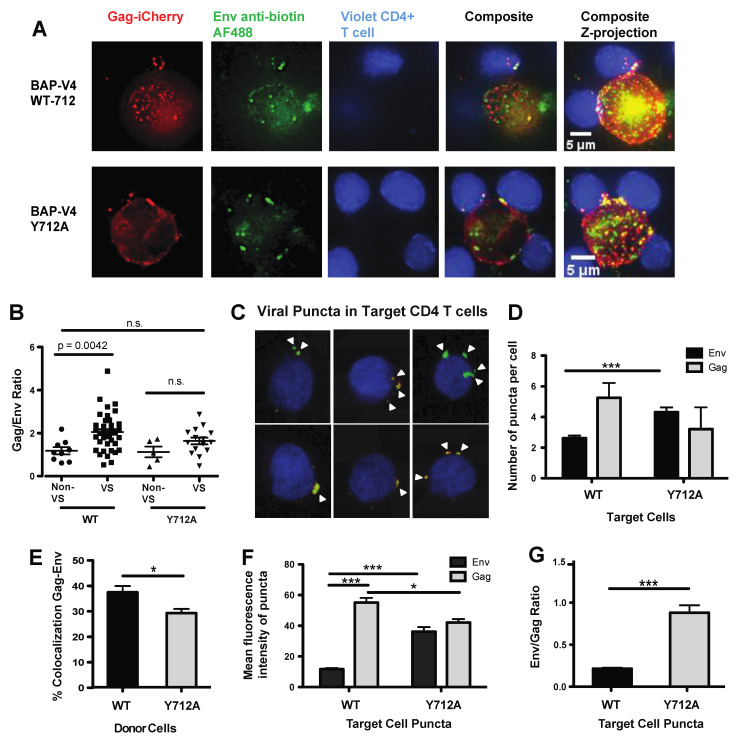
Measuring the influence of Env Y712A on the coordinated movement of Gag and Env during HIV cell–cell transfer. J-BirA cells were infected with Gag-iCherry-BAPV4 WT vs. Y712A and labeled with anti-biotin 488, and then they were co-cultured with violet dye-labeled primary CD4 target cells for 4 h to allow formation of virological synapse between the donor and target cells. Representative images taken with a Deltavision Personal DV deconvolution microscope are shown. (**A**) Gag-iCherry, Env anti-biotin, Cell Tracker Violet CD4 T cells, composite of the three images from left to right, each showing single slice of the deconvolved stack. Far right, deconvolved maximum-intensity projected images of HIV-1 cell–cell transfer depict co-localization of Gag (mCherry) and Env (AF 488) within infected cells and at VS using donor cells infected with WT (top row) or Y712A HIV-1 (bottom row). (**B**) Graph depicts the fluorescent intensity ratio biotin-Env/Gag within infected cells at non-VS sites and at VSs from segmented images of donor cells infected with WT or the Y712A mutant. (**C**) After 4 h in cell–cell transfer assays, primary CD4+ target cells were sorted for violet+ (CD4 target cells) and biotin-Env Alexa Fluor 488+ cells. Target cells imaged by confocal imaging levels of Gag (red) and Env (green) were quantified by segmentation analysis of maximum-intensity projections using Imaris software. (**D**) Graph depicts the number of puncta on target CD4 T cells that contain Env or Gag after cell–cell transfer with WT or Y712A mutant. (**E**) Graph shows the frequency of Env and Gag co-localization in target cell puncta. (**F**) Graph shows the average mean intensity of Env and Gag in target CD4 puncta. (**G**) Graph shows the Env-to-Gag intensity ratio in viral puncta on target CD4 T cells. Segmentation analysis was performed on *n* = 307 or *n* = 494 sorted primary CD4 T cells co-cultured with Gag-iCherry-BAPV4 WT- or Gag-iCherry-BAPV4 Y712A-infected J-BirA cells, respectively. The SEM of *n* = 3 experiments is shown. * *p* ≤ 0.05, *** *p* ≤ 0.001.

Next, we examined the efficiency of these endocytosis mutants to mediate HIV cell–cell transfer as compared to Env WT BAP-V4. J-BirA cells were nucleofected with BAP-V4-WT, -Y712A or -LL855AA expression vectors and used as infected donor cells in cell–cell transfer experiments with primary activated CD4 T cells as target cells. In the 4 h cell–cell transfer experiments, we observed a significant reduction in the level of Gag p24 transfer into target cells when using donor cells infected with either the Y712A or LL855AA mutant viruses (Figure 4C,D, top rows). However, in the case of the Y712A mutant, the levels of Env transfer were 2.5- to 3.5-fold higher as compared to WT and LL855AA, respectively (Figure 4C,D, bottom rows). In calculating the ratio of Env/Gag transferred into target cells, we observed a significant 4-fold increase when using Y712A-infected cells, as compared to WT- or LL855AA-infected cells. Using WT-infected cells, the relative fluorescence Env/Gag ratio was 0.26 (Figure 4A), meaning the level of Env fluorescence was 25% that of Gag fluorescence; however, using Y712A-infected cells, the levels approached a 1-to-1 Env/Gag fluorescence ratio, indicating potentially four times the Env transferred (relative to Gag) across VSs as compared to WT Env (Figure 5D; bottom graph). Paradoxically, this yielded approximately a 2-fold decrease in productive infection in target CD4 T cells 48 h post-infection (Figure 4E,F). We observed that the high levels of cell surface Env that accompany the disruption of Env endocytosis and recycling mediated by mutation of the membrane proximal tyrosine motif (Tyr 712) are still compatible with VS formation and result in the transfer of larger amounts of Env across the VS. Interestingly, the 855 dileucine mutant did not elevate surface Env relative to WT Env (Figure 4A,B), and it also did not significantly reduce the levels of HIV infection mediated through cell–cell transmission (Figure 4E,F). Surface-retained, non-recycled Y712A-Env can participate in VS formation and viral transfer, but the efficiency with which productive infection occurs in target cells under these conditions is reduced.

### 3.5. Loss of Endosomal Recycling Promotes Discoordinated Transfer of Gag and Env across VSs

We next visualized the localization of Gag and Env during VS formation and HIV-1 cell–cell transfer to gain insights into how the localization of Env may correlate with efficient cell–cell transmission. First, we used deconvolution microscopy to visualize the localization of Env and Gag in relation to VS using donor cells infected with either Env WT or Env Y712A-Gag-iCherryBAP-V4. In WT-infected cells, we observed Env within intracellular compartments and at the surface, where it was co-localized with Gag at the VS, but not highly within intracellular compartments (Figure 5A). In Y712A-infected cells, we observed a higher surface Env localization as opposed to the WT, with less Env within intracellular compartments (Figure 5A). Synapses were segmented by defining Gag accumulations at cell–cell contact sites, and Gag and Env fluorescence was determined at these sites. We then calculated the Gag-to-Env ratios within VSs of Env WT- and Env Y712A-infected cells and found a significant decrease in the Gag-to-Env fluorescence ratio in Y712A-infected cell VSs, as compared to intracellular compartments (Figure 5B).

Next, we examined the localization of Gag and Env in VS target cells after HIV-1 cell–cell transfer conducted with the WT or Y712A mutant. Jurkat + BirA cells infected with the WT or Y712A HIV Gag-iCherry-BAP-V4 were co-cultured with primary activated CD4 T cells labeled with a Far Red vital dye. After 4 h of co-culture, dye-labeled target cells containing HIV Env were sorted and analyzed using confocal microscopy and 3D segmentation analysis (Figure 5C). In the sorted CD4 target cells which had cell-associated Env or Gag puncta, we noted that cells cultured with Y712A Env-infected donor cells possessed significantly more Env-containing puncta, as compared to target cells cultured with WT Env-infected donors (Figure 5D). Moreover, we observed significantly less Env and Gag co-localization within target cells cultured with Y712A Env- vs. WT Env-infected donor cells (Figure 5E). This measure is indicative of more frequent cell-to-cell transfer of HIV Env in the absence of Gag. When examining the signal intensity of Env and Gag within co-localized puncta, we observed a 3-fold increase in the Env intensity in Y712A Env vs. WT Env puncta (Figure 5F). This was coupled with a 23% decrease in the Gag signal within Y712A vs. WT puncta (Figure 5F). Overall, this led to a 4-fold increase in the Env-to-Gag ratio detected in target cells co-cultured with HIV Y712A-infected donor cells, as compared to those co-cultured with WT HIV-infected cells (Figure 5G). In summary, the Y712A mutant resulted in less Gag and much more Env being transferred to target cells relative to the WT virus. Despite Env being transferred, the infectivity was decreased. We conclude that Env Y712 regulates the ratio of Gag and Env and promotes their ability to be co-translocated across the VS.

## 4. Discussion

The formation of VSs between infected and uninfected T cells requires the reorganization of viral and host cell proteins in both intracellular compartments and on the surface of infected cells. Previously, we utilized live cell imaging to quantify the dynamic movements of HIV Gag trafficking and reorganization during HIV cell–cell transfer [8]. We had previously observed that cell adhesion occurred prior to Gag recruitment and that once recruited, Gag accumulated at these sites of cell–cell contact between infected and uninfected T cells. Since cell adhesion requires Env, it was inferred that Env must be recruited to the synapse before Gag, and recent studies with fluorescently tagged Env have indicated that Env localization to the VS preceded Gag recruitment [12]. Here, we followed Env and Gag during and after HIV cell–cell transfer across VSs. Using an HIV carrying a V4 loop-localized BAP tag that is minimally disruptive to infection in vitro, we followed pools of Env that were labeled at the cell surface and internalized back into the cell. We identified a role for the Y712 tyrosine in coordinating the co-transfer and efficient infection across T cell VSs.

Previous studies demonstrated that the V4 region is very tolerant of small insertions without a significant loss of function [28,31]. The insertion of the 15-amino acid biotin acceptor peptide into the V4 loop of Env did not have a major impact on infectivity in cell culture and enabled utilization of biotin binding reagents. We observed no significant reduction in viral particle production, Env incorporation or virus replication (Figure 1). Further, while some tags are deleted over successive rounds of viral replication, the BAP tag was maintained during 8-day spreading infection assays (Figure 1I), suggesting that the BAP insertion does not greatly hinder viral fitness. A labeling approach that labels only a fraction of the total Env that has reached the cell surface provides a view of a protein that moves through the recycling compartment. Pulse labeling approaches can reveal how this endocytic process limits Env exposure on the cell surface yet also contributes to more efficient transmission during cell–cell transmission. Interestingly, the lack of recycling did not eliminate cell–cell infection but increased the amount of Env that was transferred while reducing cell-to-cell infectivity.

In this study, we used a dual GFP-Gag and BAP-Env construct to simultaneously track the dynamic movements of Gag and Env during HIV cell–cell transfer. Using this dual tagged virus system, we detected the transfer of both Env and Gag across VSs (Figure 3A). High-resolution fluorescence microscopy showed the accumulation and polarization of Env and Gag to conjugate interfaces, forming VSs between infected and uninfected cells (Figure 3). Additionally, we observed the accumulation of Env within intracellular compartments, which were frequently adjacent to VSs (Figure 3E). An important mechanism by which HIV evades immune detection is by maintaining low levels of Env on the surface of infected cells [21], which is achieved by the rapid endocytosis of Env after it first reaches the plasma membrane [14,38].

The major finding in these studies is that Env endocytosis is important for the transfer and transmission of HIV across VSs, though not because it is essential for Env to participate in VS formation. Our separate labels on Env and Gag allowed us to measure how relative amounts of the viral antigens vary under different conditions that were transferred and the extent to which these were efficiently copackaged during the process. We examined well-studied mutations in the membrane proximal Y712XXL and dileucine LL855 endocytosis motifs contained within the cytoplasmic tail of HIV-1 Env [17,25,39,40]. The cytoplasmic tail membrane proximal Y712XXL motif serves as a docking site for the clathrin adaptor protein AP-2 [17], and the C-terminal dileucine LL855 motif interacts with AP-1 and AP-2 [25]. These motifs have been shown to mediate endocytosis after Env’s initial trafficking to the PM [14,41]. In comparing the cell surface expression of Env, we observed that the Y712XXL but not the LL855AA CT mutation induced higher levels of surface Env expression (Figure 5A,B). The phenotypes that we observed, therefore, may implicate AP-2 and not AP-1 as being more important to the coordination of VS-mediated HIV transfer. The pronounced increase in surface Env expression was observed in the HIV Y712A Env-infected cells, which was around 6-fold higher than either WT- or 855-infected cells (Figure 4). In cell–cell transfer experiments, we observed a small but significant decrease in the efficiency of Gag transferred into target cells using donor cells infected with viruses carrying the Y712A and LL855AA mutations (Figure 4C,D). However, in the case of the Y712A mutant, we observed significantly higher levels of Env transferred into target cells (Figure 4C,D). In visualizing the localization of Env and Gag in cells engaged in VSs, we observed a higher ratio of Env- to Gag-associated fluorescence accumulating at VSs when using Y712A-infected donor cells, as compared to WT-infected cells. In contrast, we did not detect any significant changes in the biotin-Env-to-Gag fluorescence ratio within intracellular compartments (Figure 5A,B). Therefore, the increased Env at VSs in Y712A-infected cells may be attributable to the level of Env accumulated on the surface of these cells prior to engaging in cell–cell conjugates.

The increase in the Env-to-Gag ratio transferred into target cells after cell–cell HIV transfer with Y712A-infected cells correlated with a 2-fold decrease in productive infection as compared to the WT (Figure 4E,F). In infected target cells, the co-localization of Env and Gag was lower in target cells co-cultured with Y712A-infected cells vs. the WT, suggesting that the endocytic recycling pathway helps to coordinate the co-transfer of Gag with Env. As Y712 is known to be important for efficient packaging of Env onto virus particles [22], the increased fluorescent transfer of Env could be indicative of non-virion-associated Env transferred through Gag-independent pathways. Image analysis of the transferred Env/Gag puncta into target cells indicates to us that there are greater numbers of Env puncta that lack Gag. These could be related to exosome secretion and uptake and/or trogocytosis or membrane transfer that could be increased when higher levels of cell surface Env are present [42,43]. Future immunoelectron microscopy studies will be needed to confirm if Env transferred into target cells is not associated with viral particles. Taken together, the results indicate that the efficiency of HIV productive infection after HIV transfer across the VS is enhanced when HIV Env is endocytosed prior to its localization to the VS.

The cytoplasmic tail of Env is known to play important roles in regulating cell–cell transmission [34,44,45]. Recent papers have found that an increased ability of the MT4 cell line to support cell–cell transmission may be, in part, due to higher levels of surface expression of Env [46]. Although the Y712A mutant may package Env less efficiently, the higher levels of surface Env may enhance the level of Env engagement by target cells, in a manner that is independent of recycling pathways that enhance Env incorporation. Thus enhanced cell–cell interactions may compensate for decreased specific Env incorporation as it has been seen in other HIV studies [47].

Previous studies have shown that the Env Y712A motif mutations enhanced surface Env expression yet reduced the levels of Env incorporated into virus particles, thus lowering viral particle infectivity [26,48,49,50]. In contrast, the C-terminal dileucine motif enhanced the cell surface expression of Env but did not impair particle infectivity or virus replication [25]. Disrupting the Y712XXL motif also inhibited the polarized release of virions from cap-like structures at the basolateral membranes of polarized epithelial cells and in T lymphocytes (CD4 T cells) [51,52,53]. In both cases, the degree of polarization depended on the integrity of the Env cytoplasmic tail, particularly the endocytic motif Y712SPL. In a similar vein, we observed that only the 712 mutation significantly reduced productive infection in primary CD4 T cells after cell–cell transfer, while mutating the LL855 motif had no significant effect on the levels of productive infection (Figure 4E). Interestingly, mutation of the Y712XXL motif led to significantly higher levels of Env surface expression as compared to the LL855 motif mutant (Figure 4A,B), suggesting that the 712 motif serves to internalize the majority of Env at the surface of infected cells. The fact that the 712 mutant resulted in the discoordinated transfer of Gag and Env across VSs (Figure 4C,D) further leads us to suggest that a lack of Env endocytosis leads to inefficient coupling of Env incorporation with Gag assembly. Other studies have demonstrated that cell–cell transmission requires the production of infectious enveloped particles, and that the truncation of the CT tail leads to approximately a 2-fold inhibition of productive infection in target cells [54]. Taken together, these data support a model whereby Env recycling enhances the efficiency by which Gag and Env come together at VSs and assemble into virus particles prior to being transferred into permissive target cells.

Previous studies have demonstrated that the CT-dependent incorporation of Env into virus particles is cell type-dependent, implying that host factors may interact with the CT of HIV in certain cell types to mediate efficient incorporation into budding viral particles [54]. Indeed, the role of the Rab11-interacting protein FIP1C in Env packaging has been shown to be a cell type-dependent factor that is involved in mediating the endosomal trafficking of Env to sites of cell-free virus assembly [22]. The retromer, a protein complex involved in retrograde transport from the endosome to the Golgi, is also required for efficient Env packaging, further implicating recycling pathways in efficient viral infection [24]. It is likely that the pathways that mediate cell-free and cell–cell virus assembly overlap, and that the VS provides a further spatial constraint on where virus assembly occurs. Further study of this pathway will identify novel targets for rational drug design to inhibit this potent mode of HIV transmission.

Currently, there are several non-exclusive models of Env incorporation into cell-free virus particles that include passive incorporation, direct Gag–Env interactions, Gag–Env co-targeting and indirect Gag–Env interaction models [14,55]. Studies examining cell-free virus particle assembly demonstrate that the Rab11 family-interacting protein 1C (FIP1C) directs Env incorporation into budding particles in an HIV cytoplasmic tail-dependent manner [22,23]. Our studies are consistent with models whereby Env incorporation during VS formation is also dependent on Env being first endocytosed and then sorted from endosomal recycling compartments prior to becoming incorporated into budding virions.

## Data Availability

Data is contained within the article or supplementary material.

## Supplementary Materials

The following are available online at https://www.mdpi.com/article/10.3390/v13091729/s1, Movie S1: Anti-biotin staining allows envelope visualization using spinning disk confocal microscopy performed on a Zeiss Axio Observer Z1, Yokogawa spinning disk confocal microscope. HIV Gag-iCerulean-BAP was stained at 37 °C for 1 h with anti-biotin Alexa Fluor 488, and after washing, the cells were incubated with primary human CD4 T cells previously labeled with Far Red (Invitrogen). Gain = 150, Exp = 80 ms, 3D images were acquired every 4–5 s, with 150 total timepoints acquired over approximately 10 min. 20 z planes were acquired for each 3D image, with a spacing of 0.75 um between planes. Supplemental Figure S1: Western blot of HIV BAP-V4 transfected 293T cells without and with addition of a 10:1 ratio of HIV BAP-V4: BirA plasmid. HIV polyclonal sera is used to detect Env gp160 and gp120, and Gag preccursor p55 as well as processed p24 CA.

## References

[1] Jolly C., Kashefi K., Hollinshead M., Sattentau Q.J. (2004). HIV-1 cell to cell transfer across an Env-induced, actin-dependent synapse. J. Exp. Med..

[2] Dimitrov D.S., Willey R.L., Sato H., Chang L.J., Blumenthal R., Martin M.A. (1993). Quantitation of human immunodeficiency virus type 1 infection kinetics. J. Virol..

[3] Chen P., Hubner W., Spinelli M.A., Chen B.K. (2007). Predominant mode of human immunodeficiency virus transfer between T cells is mediated by sustained Env-dependent neutralization-resistant virological synapses. J. Virol..

[4] Agosto L.M., Uchil P.D., Mothes W. (2015). HIV cell-to-cell transmission: Effects on pathogenesis and antiretroviral therapy. Trends Microbiol..

[5] Alvarez R.A., Barria M.I., Chen B.K. (2014). Unique features of HIV-1 spread through T cell virological synapses. PLoS Pathog..

[6] Jolly C., Sattentau Q.J. (2004). Retroviral spread by induction of virological synapses. Traffic.

[7] Sigal A., Kim J.T., Balazs A.B., Dekel E., Mayo A., Milo R., Baltimore D. (2011). Cell-to-cell spread of HIV permits ongoing replication despite antiretroviral therapy. Nature.

[8] Hubner W., McNerney G.P., Chen P., Dale B.M., Gordon R.E., Chuang F.Y., Li X.D., Asmuth D.M., Huser T., Chen B.K. (2009). Quantitative 3D video microscopy of HIV transfer across T cell virological synapses. Science.

[9] Jin J., Sherer N.M., Heidecker G., Derse D., Mothes W. (2009). Assembly of the murine leukemia virus is directed towards sites of cell-cell contact. PLoS Biol..

[10] Park R.J., Wang T., Koundakjian D., Hultquist J.F., Lamothe-Molina P., Monel B., Schumann K., Yu H., Krupzcak K.M., Garcia-Beltran W. (2017). A genome-wide CRISPR screen identifies a restricted set of HIV host dependency factors. Nat Genet.

[11] Starling S., Jolly C. (2016). LFA-1 engagement triggers T cell polarization at the HIV-1 virological synapse. J. Virol..

[12] Wang L., Izadmehr S., Kamau E., Kong X.P., Chen B.K. (2019). Sequential trafficking of Env and Gag to HIV-1 T cell virological synapses revealed by live imaging. Retrovirology.

[13] Murakami T. (2008). Roles of the interactions between Env and Gag proteins in the HIV-1 replication cycle. Microbiol Immunol.

[14] Checkley M.A., Luttge B.G., Freed E.O. (2011). HIV-1 envelope glycoprotein biosynthesis, trafficking, and incorporation. J. Mol. Biol..

[15] Murphy R.E., Saad J.S. (2020). The interplay between HIV-1 gag binding to the plasma membrane and env incorporation. Viruses.

[16] Tedbury P.R., Freed E.O. (2014). The role of matrix in HIV-1 envelope glycoprotein incorporation. Trends Microbiol..

[17] Boge M., Wyss S., Bonifacino J.S., Thali M. (1998). A membrane-proximal tyrosine-based signal mediates internalization of the HIV-1 envelope glycoprotein via interaction with the AP-2 clathrin adaptor. J. Biol. Chem..

[18] Egan M.A., Carruth L.M., Rowell J.F., Yu X., Siliciano R.F. (1996). Human immunodeficiency virus type 1 envelope protein endocytosis mediated by a highly conserved intrinsic internalization signal in the cytoplasmic domain of gp41 is suppressed in the presence of the Pr55gag precursor protein. J. Virol..

[19] Kirschman J., Qi M., Ding L., Hammonds J., Dienger-Stambaugh K., Wang J.J., Lapierre L.A., Goldenring J.R., Spearman P. (2018). HIV-1 envelope glycoprotein trafficking through the endosomal recycling compartment is required for particle incorporation. J. Virol..

[20] Hogan M.J., Conde-Motter A., Jordan A.P.O., Yang L., Cleveland B., Guo W., Romano J., Ni H., Pardi N., LaBranche C.C. (2018). Increased surface expression of HIV-1 envelope is associated with improved antibody response in vaccinia prime/protein boost immunization. Virology.

[21] von Bredow B., Arias J.F., Heyer L.N., Gardner M.R., Farzan M., Rakasz E.G., Evans D.T. (2015). Envelope glycoprotein internalization protects human and simian immunodeficiency virus-infected cells from antibody-dependent cell-mediated cytotoxicity. J. Virol..

[22] Qi M., Chu H., Chen X., Choi J., Wen X., Hammonds J., Ding L., Hunter E., Spearman P. (2015). A tyrosine-based motif in the HIV-1 envelope glycoprotein tail mediates cell-type- and Rab11-FIP1C-dependent incorporation into virions. Proc. Natl. Acad. Sci. USA.

[23] Qi M., Williams J.A., Chu H., Chen X., Wang J.J., Ding L., Akhirome E., Wen X., Lapierre L.A., Goldenring J.R. (2013). Rab11-FIP1C and Rab14 direct plasma membrane sorting and particle incorporation of the HIV-1 envelope glycoprotein complex. PLoS Pathog..

[24] Groppelli E., Len A.C., Granger L.A., Jolly C. (2014). Retromer regulates HIV-1 envelope glycoprotein trafficking and incorporation into virions. PLoS Pathog..

[25] Wyss S., Berlioz-Torrent C., Boge M., Blot G., Honing S., Benarous R., Thali M. (2001). The highly conserved C-terminal dileucine motif in the cytosolic domain of the human immunodeficiency virus type 1 envelope glycoprotein is critical for its association with the AP-1 clathrin adaptor [correction of adapter]. J. Virol..

[26] Bhakta S.J., Shang L., Prince J.L., Claiborne D.T., Hunter E. (2011). Mutagenesis of tyrosine and di-leucine motifs in the HIV-1 envelope cytoplasmic domain results in a loss of Env-mediated fusion and infectivity. Retrovirology.

[27] Tannous B.A., Grimm J., Perry K.F., Chen J.W., Weissleder R., Breakefield X.O. (2006). Metabolic biotinylation of cell surface receptors for in vivo imaging. Nat. Methods.

[28] Ren X., Sodroski J., Yang X. (2005). An unrelated monoclonal antibody neutralizes human immunodeficiency virus type 1 by binding to an artificial epitope engineered in a functionally neutral region of the viral envelope glycoproteins. J. Virol..

[29] Munro J.B., Gorman J., Ma X., Zhou Z., Arthos J., Burton D.R., Koff W.C., Courter J.R., Smith A.B., Kwong P.D. (2014). Conformational dynamics of single HIV-1 envelope trimers on the surface of native virions. Science.

[30] Adachi A., Gendelman H.E., Koenig S., Folks T., Willey R., Rabson A., Martin M.A. (1986). Production of acquired immunodeficiency syndrome-associated retrovirus in human and nonhuman cells transfected with an infectious molecular clone. J. Virol..

[31] Pantophlet R., Wang M., Aguilar-Sino R.O., Burton D.R. (2009). The human immunodeficiency virus type 1 envelope spike of primary viruses can suppress antibody access to variable regions. J. Virol..

[32] Sherer N.M., Lehmann M.J., Jimenez-Soto L.F., Ingmundson A., Horner S.M., Cicchetti G., Allen P.G., Pypaert M., Cunningham J.M., Mothes W. (2003). Visualization of retroviral replication in living cells reveals budding into multivesicular bodies. Traffic.

[33] Pear W.S., Nolan G.P., Scott M.L., Baltimore D. (1993). Production of high-titer helper-free retroviruses by transient transfection. Proc. Natl. Acad. Sci. USA.

[34] Durham N.D., Chen B.K. (2015). HIV-1 cell-free and cell-to-cell infections are differentially regulated by distinct determinants in the env gp41 cytoplasmic tail. J. Virol..

[35] Burton D.R., Barbas C.F., Persson M.A., Koenig S., Chanock R.M., Lerner R.A. (1991). A large array of human monoclonal antibodies to type 1 human immunodeficiency virus from combinatorial libraries of asymptomatic seropositive individuals. Proc. Natl. Acad. Sci. USA.

[36] Niers J.M., Chen J.W., Weissleder R., Tannous B.A. (2011). Enhanced in vivo imaging of metabolically biotinylated cell surface reporters. Anal. Chem..

[37] Durham N.D., Chen B.K. (2016). Measuring T cell-to-T cell HIV-1 transfer, viral fusion, and infection using flow cytometry. Methods Mol. Biol..

[38] Egan M.A., Carruth L.M., Rowell J.F., Yu X., Siliciano R.F. (1997). The ins and outs of HIV endocytosis. Trends Cell Biol..

[39] Ohno H., Aguilar R.C., Fournier M.C., Hennecke S., Cosson P., Bonifacino J.S. (1997). Interaction of endocytic signals from the HIV-1 envelope glycoprotein complex with members of the adaptor medium chain family. Virology.

[40] Rowell J.F., Stanhope P.E., Siliciano R.F. (1995). Endocytosis of endogenously synthesized HIV-1 envelope protein. Mechanism and role in processing for association with class II MHC. J. Immunol..

[41] Byland R., Vance P.J., Hoxie J.A., Marsh M. (2007). A conserved dileucine motif mediates clathrin and AP-2-dependent endocytosis of the HIV-1 envelope protein. Mol. Biol. Cell.

[42] Arakelyan A., Fitzgerald W., Zicari S., Vanpouille C., Margolis L. (2017). Extracellular vesicles carry HIV env and facilitate Hiv infection of human lymphoid tissue. Sci. Rep..

[43] Bastos-Amador P., Perez-Cabezas B., Izquierdo-Useros N., Puertas M.C., Martinez-Picado J., Pujol-Borrell R., Naranjo-Gomez M., Borras F.E. (2012). Capture of cell-derived microvesicles (exosomes and apoptotic bodies) by human plasmacytoid dendritic cells. J. Leukoc. Biol..

[44] Emerson V., Haller C., Pfeiffer T., Fackler O.T., Bosch V. (2010). Role of the C-terminal domain of the HIV-1 glycoprotein in cell-to-cell viral transmission between T lymphocytes. Retrovirology.

[45] Fernandez M.V., Freed E.O. (2018). Meeting review: 2018 International Workshop on Structure and Function of the Lentiviral gp41 Cytoplasmic Tail. Viruses.

[46] Fernandez M.V., Hoffman H.K., Pezeshkian N., Tedbury P.R., van Engelenburg S.B., Freed E.O. (2020). Elucidating the basis for permissivity of the MT-4 T-cell line to replication of an HIV-1 mutant lacking the gp41 cytoplasmic tail. J. Virol..

[47] Van Duyne R., Kuo L.S., Pham P., Fujii K., Freed E.O. (2019). Mutations in the HIV-1 envelope glycoprotein can broadly rescue blocks at multiple steps in the virus replication cycle. Proc. Natl. Acad. Sci. USA.

[48] Day J.R., Munk C., Guatelli J.C. (2004). The membrane-proximal tyrosine-based sorting signal of human immunodeficiency virus type 1 gp41 is required for optimal viral infectivity. J. Virol..

[49] Lambele M., Labrosse B., Roch E., Moreau A., Verrier B., Barin F., Roingeard P., Mammano F., Brand D. (2007). Impact of natural polymorphism within the gp41 cytoplasmic tail of human immunodeficiency virus type 1 on the intracellular distribution of envelope glycoproteins and viral assembly. J. Virol..

[50] West J.T., Weldon S.K., Wyss S., Lin X., Yu Q., Thali M., Hunter E. (2002). Mutation of the dominant endocytosis motif in human immunodeficiency virus type 1 gp41 can complement matrix mutations without increasing Env incorporation. J. Virol..

[51] Deschambeault J., Lalonde J.P., Cervantes-Acosta G., Lodge R., Cohen E.A., Lemay G. (1999). Polarized human immunodeficiency virus budding in lymphocytes involves a tyrosine-based signal and favors cell-to-cell viral transmission. J. Virol..

[52] Lodge R., Gottlinger H., Gabuzda D., Cohen E.A., Lemay G. (1994). The intracytoplasmic domain of gp41 mediates polarized budding of human immunodeficiency virus type 1 in MDCK cells. J. Virol..

[53] Lodge R., Lalonde J.P., Lemay G., Cohen E.A. (1997). The membrane-proximal intracytoplasmic tyrosine residue of HIV-1 envelope glycoprotein is critical for basolateral targeting of viral budding in MDCK cells. EMBO J..

[54] Monel B., Beaumont E., Vendrame D., Schwartz O., Brand D., Mammano F. (2012). HIV cell-to-cell transmission requires the production of infectious virus particles and does not proceed through env-mediated fusion pores. J. Virol..

[55] Johnson M.C. (2011). Mechanisms for Env glycoprotein acquisition by retroviruses. AIDS Res. Hum. Retrovir..

